# Thermodynamic modeling of transcription: sensitivity analysis differentiates biological mechanism from mathematical model-induced effects

**DOI:** 10.1186/1752-0509-4-142

**Published:** 2010-10-24

**Authors:** Jacqueline M Dresch, Xiaozhou Liu, David N Arnosti, Ahmet Ay

**Affiliations:** 1 Department of Mathematics, Michigan State University, East Lansing, MI, USA; 2 Department of Biochemistry and Molecular Biology, Michigan State University, East Lansing, MI, USA; 3 Department of Biology, Colgate University, Hamilton, NY, USA; 4 Department of Mathematics, Colgate University, Hamilton, NY, USA

## Abstract

**Background:**

Quantitative models of gene expression generate parameter values that can shed light on biological features such as transcription factor activity, cooperativity, and local effects of repressors. An important element in such investigations is sensitivity analysis, which determines how strongly a model's output reacts to variations in parameter values. Parameters of low sensitivity may not be accurately estimated, leading to unwarranted conclusions. Low sensitivity may reflect the nature of the biological data, or it may be a result of the model structure. Here, we focus on the analysis of thermodynamic models, which have been used extensively to analyze gene transcription. Extracted parameter values have been interpreted biologically, but until now little attention has been given to parameter sensitivity in this context.

**Results:**

We apply local and global sensitivity analyses to two recent transcriptional models to determine the sensitivity of individual parameters. We show that in one case, values for repressor efficiencies are very sensitive, while values for protein cooperativities are not, and provide insights on why these differential sensitivities stem from both biological effects and the structure of the applied models. In a second case, we demonstrate that parameters that were thought to prove the system's dependence on activator-activator cooperativity are relatively insensitive. We show that there are numerous parameter sets that do not satisfy the relationships proferred as the optimal solutions, indicating that structural differences between the two types of transcriptional enhancers analyzed may not be as simple as altered activator cooperativity.

**Conclusions:**

Our results emphasize the need for sensitivity analysis to examine model construction and forms of biological data used for modeling transcriptional processes, in order to determine the significance of estimated parameter values for thermodynamic models. Knowledge of parameter sensitivities can provide the necessary context to determine how modeling results should be interpreted in biological systems.

## Background

Mathematical modeling of gene transcription is becoming a common approach to gain insight into the physical and chemical properties that drive transcription and to characterize the nature of gene networks that form the basis of biological systems [1-9]. By formulating the transcriptional process quantitatively, one can derive parameter values that highlight important features of gene regulation, including protein-protein interactions, protein-DNA interactions, and their effects on gene expression. Major types of models in use include Boolean, ordinary differential equation (ODE), and thermodynamic; these models employ parameters such as synthesis, decay, and diffusion rates for proteins and mRNA, as well as binding affinity, repression efficiency, and cooperativity of transcription factors [1-9]. Below, we focus on analysis of thermodynamic models, which unlike other types of models, specifically consider the DNA sequence in transcriptional control regions. For all of these models, parameters are usually not known and the derivation of these parameters from biological data, such as images from confocal microscopy, is complicated by the noisy nature of biological data. Two major challenges then face the modeler who seeks a biological interpretation of the parameter values. First, large parameter ranges are often found, leading to uncertainty about where realistic parameter values lie. In many cases, relevant experimental measurements have not been conducted - mathematical models are often in fact the best method to determine such values. Second, parameter values can be strongly influenced by the form used to shape the problem, thus the problem then becomes whether these extracted values are realistic - are the values due to properties of the biological system or the mathematical model?

To address these questions, one must examine the mathematical model itself to determine the uncertainty in parameter values and ascertain the impact that perturbations in parameter values will have on the model output. This question of parameter uncertainty and sensitivity analysis has been addressed in many different applications, including biological, chemical and risk assessment [10-16]. Parameter uncertainties have been explored extensively for ODE models, which typically have a very large number of parameters, resulting in a great deal of model variation and parameter uncertainty [10-13]. The sensitivity of parameters derived from thermodynamic transcription models, however, has not yet been examined in such a context; studies have focused simply on extracting and interpreting parameter values or parameter ranges [1-4,6].

Thermodynamic models, also termed fractional occupancy models, are based on formulations originating from statistical physics. For transcriptional analysis, these models consider all possible states of a DNA regulatory element, where a state refers to a specific configuration of regulatory proteins (transcription factors) bound to DNA [1-4,6]. Each state is awarded a weight, which depends on properties such as the binding affinity and concentration of proteins. In many thermodynamic models, including those examined in this study, the mathematical formula used to calculate the expression level is a rational function in which parameters to be estimated can be found in both the numerator and denominator [1,2].

**Figure 1:**
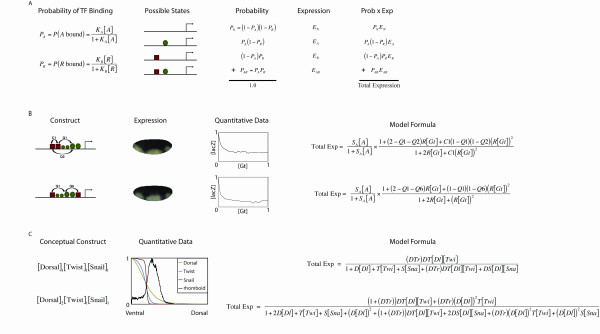
**General description of thermodynamic models and examples of constructs, data sets, and formulas used for the models of Zinzen et al. and Fakhouri et al**. A) Thermodynamic models. The first column shows the probability of a transcription factor binding. Square brackets denote protein concentrations and *K *values represent binding parameters. The second column illustrates all possible states of an enhancer containing one repressor and one activator binding site, the third the probability of each occurring and the fourth the expression contribution for each. The last column shows how the total expression is calculated, as a sum of each states' probability multiplied by its expression contribution. B) Examples of the implementation of the Fakhouri et al. model. The first column gives examples of two constructs used, the second representative embryos from each, imaged for *lacZ *reporter gene activity, the third the actual normalized repressor [Gt] versus reporter gene [*lacZ*] levels used for modeling, and the last the model's total expression formulas. For sensitivity analysis, the RMSE is calculated using the model formula for each of the twelve constructs; this figure shows only two examples (other constructs described in [1]). C) Examples of the implementation of the Zinzen et al. model. The first column lists two conceptual constructs used. These are not depicted as in A) and B) because the Zinzen et al. model does not consider order or spacing of binding sites. Enhancers are described solely by the number of binding sites. Terms inside brackets indicate the presence of binding sites for that protein, while subscripts indicate the number of binding sites. The second column contains quantitative data of normalized [Dl], [Twi], and [Sna] proteins and [*rho*] mRNA used for modeling the *rho *enhancer. The last column gives the model's total expression formulas for each of these conceptual constructs.

Sensitivity analysis can be applied to any field that uses mathematical modeling as a tool, and has been used extensively on diverse models in economics, civil engineering, and medicine [17-19]. In drug design, sensitivity analysis has been used in determining which parameter or parameters of a differential equation model would have the largest effect on a given outcome of interest, such as the sugar level in the blood of a patient [19]. We show here its utility when applied to thermodynamic models of gene regulation (Figure 1). Such models have been applied to systems ranging from a single cis-regulatory element to diverse collections of highly divergent elements bound by a wide range of proteins [3,6]. Here, we concentrate on two studies that took a very focused approach to identify specific features relating to transcriptional activation, repression and cooperativity by examining sets of similar enhancers that feature a limited degree of variation.

## Methods

Sensitivity analysis, whether local or global, has the primary goal of determining how a given model responds to variations in parameter values. Local sensitivity analysis (also known as differential analysis or nominal range sensitivity analysis) focuses on a particular point in parameter space, varying parameters one at a time to obtain a local response of the model to each parameter [13,15,20,21]. Global sensitivity analysis, on the other hand, tries to capture the entire parameter space all at once, allowing multiple parameter values to be explored simultaneously. Parameters are individually analyzed by averaging the variation in model output over the entire space [20]. It is possible to explore multiparameter responses using local sensitivity analysis, employing techniques such as second-order partial derivatives, but this analysis can be computationally costly. For this reason, in this study we use global sensitivity analysis to investigate multiparameter effects.

Our approach initially used local sensitivity analysis to calculate the model's rate of change with respect to each variable at a given point in parameter space, quantifying at that point how small changes in each variable will affect the model output. Then, we turned to global analysis, employing three different methods. The first simply takes an average of results obtained from local sensitivity analyses on different points in parameter space. The other two global methods each rely on determining which parameters and parameter combinations contribute most to the variation in the model output that resulted from sampling a wide range of parameter values. The eFAST algorithm does this through Fourier analysis, while the HDMR algorithm does this through polynomial approximation. For more details on each individual method, see the following text and the model descriptions and formulas found in the Additional file 1.

### Local Sensitivity Analysis

Most local sensitivity analysis methods use partial derivatives evaluated at a point in parameter space to determine how the model output changes locally with respect to small variations of a particular parameter [11,13,21,22]. To gain insight on local effects, when calculating the sensitivity coefficient, the partial derivative is normalized using the relative changes in quantities [20,22]. To calculate the local sensitivity coefficient for an objective function, *C*, with respect to a parameter, *μ*, one uses the formula [22]:

$$\text{sensitivity coefficient}=\frac{\mu }{C}\left(\frac{\partial }{\partial \mu }C\right)$$

A major advantage of local sensitivity analysis is its ease of implementation [21]. The formula is straightforward and the results are simple to interpret, but must be viewed with caution, because the analysis is only valid in the small region around a particular point [11,12,14]. Also, this method does not account for parameter interactions, so in non-linear models, this often results in an underestimation of true model sensitivities [15,21]. In many mathematical models of biological events, including differential equation models of the gap gene network in Drosophila, parameter ranges are fairly well constrained due to previous studies which have estimated values for parameters such as diffusion and decay rates [23-25]. This allows a great deal of information to be extracted from a local analysis. With the large uncertainty of parameter values and ranges in models such as those we focus on in this study, this method is not robust, so global sensitivity techniques are preferred.

### Global Sensitivity Analysis

These approaches vary parameters over a larger parameter space and have the ability to quantify parameter interactions [12,14,15,21]. Global sensitivity analysis covers a much wider range of methods than that of local sensitivity analysis. A common element is that global sensitivity methods explore the full parameter space, quantifying a model's sensitivity to perturbations in each parameter. Due to computational limitations, this exploration of parameter space requires a sampling method that randomly samples parameter space, while evenly covering individual parameter ranges [13]. Here, we focus on three global sensitivity methods: a basic, easily-implemented method of average local sensitivities, and two more complex methods that capture first-order sensitivities, as well as higher order sensitivities, those sensitivities derived from varying more than one parameter. Higher order sensitivities can capture possible parameter interactions. An example of higher order interactions would be the relationship between the intrinsic activity of a transcription factor and cooperative binding. Solutions to a model would include those that ascribed high cooperativity between weak neighboring transcription factors, as well as low cooperativity between inherently powerful transcription factors. Thus, the higher order sensitivity would quantify the impact this relationship has on the model output. For a particular system, varying cooperativity or transcription factor activity alone may have little effect on model output, thus these parameters would have small first-order sensitivity indices, because the two effects can "cancel each other out" or compensate. If cooperativity and activity are simultaneously varied, large effects might be observed, producing significant second-order sensitivities.

#### i) Method 1

To overcome the major disadvantage of the small region of sampling in local sensitivity analysis while retaining the ease of computational simplicity, some studies have used a global approach of averaging local sensitivities taken throughout the parameter space [13]. This method chooses sets of parameters randomly using realistic ranges of each parameter space, then local sensitivity analysis is applied to each set of parameters, and results are averaged over the entire space for each parameter to provide a global sensitivity coefficient. This method can be computationally expensive.

#### ii) Method 2

One of the earliest, yet most efficient global sensitivity analysis methods is the variance-based Fourier amplitude sensitivity test (FAST) [26]. FAST uses a unique search-curve to explore parameter space and employs Fourier decomposition of the objective function to calculate main effects, or first-order sensitivity indices [15,20]. The FAST method does not rely on any assumptions about the form of the model, thus it can be used on nonlinear, monotonic or nonmonotonic models [15,20]. An extension of the FAST method (eFAST) can compute not only the first-order, but also the total-order sensitivity coefficients for a given parameter, as defined by Sobol' [12,20]:

$$S_{T_{i}}=S_{i}+\sum_{\begin{array}{l} j=1\\ j\neq i \end{array}}^{n}S_{ij}+\sum_{\begin{array}{l} 1\leq j<k\leq n\\ j\neq i,k\neq i \end{array}}S_{ijk}+\ldots +S_{12...n}$$

The search-curve used to sample the parameter space is defined by a collection of sinusoidal functions, one for each parameter. The assigned frequencies of these functions are then used to partition the variance amongst parameters and calculate the first-order sensitivity indices by Fourier analysis. For each parameter, the eFAST algorithm calculates the first order sensitivity of that parameter as well as the first order sensitivity of the entire set of remaining parameters. The total-order sensitivity coefficient for the parameter of interest is then approximated by the sum of that parameter's first-order sensitivity and the remaining variance after first-order partitioning. (See Additional file 1 for mathematical formulas.)

#### iii) Method 3

Another global sensitivity analysis method, ANalysis Of Variance (ANOVA), decomposes a function into a summation of terms of increasing dimensionality [14]:

$$f\left(x\right)=f_{0}+\sum_{i=1}^{n}f_{i}\left(x_{i}\right)+\sum_{1\leq i<j\leq n}f_{ij}\left(x_{i},x_{j}\right)+...+f_{12...n}\left(x_{1},x_{2},...,x_{n}\right)$$

where *f*_0 _is the main effect (also designated the zeroth order term, or the overall mean) [14,21]. Note that this method is distinct from the ANOVA commonly used in biological studies when comparing two or more groups of experimental data. We will refer to ANOVA in this study as the high dimensional model representation (HDMR) expansion [14]. The underlying assumption for an HDMR method is that the objective function is normally distributed [14,15,21]. Due to random sampling, this is typically not violated, but if a violation occurs, corrective measures can be taken [15]. Previous studies have shown that, when implemented, first and second-order effect terms are typically sufficient for a good approximation of the total sensitivity [27,28]. In calculating the terms in the expansion, the Monte Carlo integration method is used [14]. The HDMR algorithm used in this study is computationally highly efficient due to the use of orthonormal polynomial approximations for first and second-order terms [14]. These terms are then normalized by the total variance to obtain first and second-order sensitivity indices corresponding to the main effect of each parameter, and the effects from pair-wise parameter interactions [14]. (See Additional file 1 for mathematical formulas.)

## Results

To determine parameter sensitivities, we analyzed two different thermodynamic models recently used to study transcriptional regulation in the early *Drosophila *embryo; neither publication included a thorough sensitivity analysis of the parameters. In the first work, Arnosti and colleagues extracted their model parameters using protein and mRNA concentration data from a set of simplified regulatory elements functionally tested in embryos [Figure 1; [1]]. This model was implemented using 9 different variations, with the total number of parameters ranging from 8 to 13. The parameters represent the overall repressor activity (R), distance-function for repression (Q), and homotypic repressor cooperativities (C). In the second model, Levine and colleagues extracted their model parameters using protein and mRNA concentration data representing the activity of two endogenous regulatory elements [2]. Their model analyzes the expected activity of 128 different hypothetical regulatory sequences, with 9 different parameters for each element representing protein binding affinities, and homotypic and heterotypic cooperativities. In this study, we focused on the 8 forms that produced the best correlation with the experimental data, published in Table S2 of Zinzen et al..

### Local Sensitivity Analysis of the Fakhouri et al. Model

To initiate our study, we performed the local sensitivity analysis on the model of Fakhouri et al. In this study, Arnosti and colleagues pursued a unique "bottom up" approach to understand the mechanistic processing of regulatory elements by the transcriptional machinery, using a well defined and characterized set of repressors and activators in *Drosophila *blastoderm embryos. Their approach not only incorporated cooperativity between transcription factors and a quenching activity of repressor proteins, as other models have done [2-9], but included distance dependence and relative enhancer positioning of these activities with no a priori assumptions. In contrast to earlier enhancer based studies, which use many bioinformatically determined binding sites, they created and systematically analyzed a well-defined set of transcriptional regulatory elements in the *Drosophila *embryo with experimentally determined binding sites, focusing on repressor-activator spacing, stoichiometry and arrangement. Due to this well defined construct design, their data set also depends on a fewer number of features than previous studies. They used experimental data from 12 of these elements to extract parameter values representing repressor scaling factor, efficiencies, and cooperativities. Fakhouri et al. used the root mean square error (RMSE) to determine the quality of fit, thus, for the sensitivity analysis of this model, RMSE was used as the objective function [1]. Their study employed nine model schemes, which differ slightly in their treatment of distance effects and cooperativity. Each scheme uses the same general formula as the equations shown in Figure 1B, but the treatment of distance-dependent repression and cooperativity differs. In different schemes, repressor-activator distances were "binned" into different intervals, (e.g. 0-6 bp, 28-41 bp, 50-56 bp etc. vs. 0-6 bp, 28-31 bp, 41-50 bp etc.) and cooperativity was represented as a single term or two different terms for genes containing three repressor binding sites, resulting a in different number of parameters for each scheme. For a full description of all nine schemes, refer to [1].

**Figure 2:**
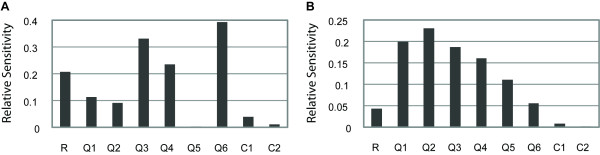
**Sensitivity analysis using local and global-local methods on the thermodynamic model of Fakhouri et al., scheme 2**. A) Local sensitivity analysis highlights great differences among parameters. Model parameters are shown on the horizontal axis and local sensitivity coefficients (scaled from 0.0 to 1.0) are shown on the vertical axis. Larger values indicate that the model output changes greatly when this parameter is varied. B) Global-local analysis reveals differences in relative sensitivities compared to local analysis. The global-local sensitivity contribution (also scaled from 0.0 to 1.0) is plotted on the vertical axis. In the global-local analysis, quenching parameters (Q) are generally more sensitive than repressor scaling factors (R), which in turn are more sensitive than cooperativity parameters (C).

We analyzed the local sensitivity of the Fakhouri et al. model at parameter values optimized in their study using scheme 2 (Figure 2A). To reduce the dependence of local analysis on a single set of optimized values, we applied local sensitivity analysis in a global fashion. 10,000 sets of parameter values within biologically reasonable boundaries were generated, and for each parameter, a weighted average sensitivity coefficient was then calculated. We found that this global/local approach resulted in sensitivity coefficients which differ significantly from the local sensitivities found using the optimized values (Figures 2B and S1). This difference is expected, because local sampling of parameter space may not reflect the overall possible sensitivities of all parameter values. The sensitivities converge to those identified by global methods, as noted below, and reveal significant differences among the parameters' sensitivities.

The global/local approach avoids the problem of dependence on estimated parameter values, and is global in the sense that the whole parameter space is sampled, but it fails to capture higher-level interactions of parameters. Therefore, we applied global analyses to test this model using two well-established methods.

### Global Sensitivity Analysis of the Fakhouri et al. Model

**Figure 3:**
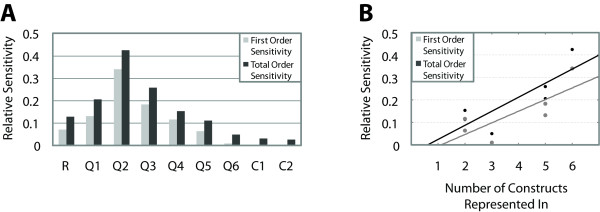
**Sensitivity analysis using the eFAST global sensitivity method on the thermodynamic model of Fakhouri et al., scheme 2**. A) Shown are first- and total-order sensitivity indices, which represent the amount of variation in the model output with respect to each parameter individually (gray bars) and in conjunction with all other parameters (black bars), scaled from 0.0 to 1.0. If the gray bar is much smaller than the black bar, as in Q6, secondary (or higher) effects are predominant. Quenching parameters are generally most sensitive and cooperativity parameters are least sensitive. B) Effect of frequency with which a quenching parameter is represented in the twelve constructs on sensitivity. The number of constructs that the parameter is represented in is shown along the horizontal axis. Corresponding first- and total-order sensitivity indices are shown for each quenching parameter, as calculated by the eFAST algorithm. There are two quenching parameters (Q4 and Q5) represented in 2 constructs, and two quenching parameters (Q1 and Q3) represented in 5 constructs. At these values, there are four data points, two for each quenching parameter. The lines illustrate linear fits to each data set, first- and total-order sensitivity indices. In general, the more constructs a quenching parameter is represented in, the more sensitive that parameter.

We applied the eFAST global sensitivity analysis method to the nine formulations of the model of Fakhouri et al. and obtained comparable results with all of them (Figures 3 and S2). Significantly, we found that the quenching parameters, those that represent distance-dependent repression, are generally the most sensitive and the repressor-repressor cooperativity parameters are the least sensitive. This general trend was observed with all nine schemes (Figures 3 and S2). The Fakhouri et al. data set was constructed to specifically test spacing effects, i.e. distance-dependent quenching of activators by short-range repressors, and this feature was thoroughly explored by a large number of gene constructs. Thus, it is reasonable that with this particular experimental data used to fit the parameters, in which quenching terms are represented in many constructs, the quenching parameters are more sensitive than other parameters because differences in fit quality will reflect contributions to the RMSE from many constructs. A small change will be magnified because it contributes to a larger number of the terms in the RMSE. We tested whether the number of gene constructs in which each parameter is represented affects that parameter sensitivity for other features (Figure 3B). We noted that in general, the more gene constructs in which a quenching parameter is represented, the more sensitive that parameter. The frequency with which a feature is found is not the sole influence on sensitivity, however. Some quenching parameters, such as Q1 and Q3, are seen in the same number of constructs but have different sensitivity indices. We hypothesize that this difference is due to parameter interactions, and test this idea as described below.

From a strictly mathematical viewpoint, the enhanced sensitivity of quenching parameters over cooperativity parameters is also not surprising. The form of this model itself suggests that small changes in quenching parameters will have the largest effects on the model output, because quenching parameters appear only in the numerator of the expression function, while the repressor scaling factor and cooperativity parameters of the same form appear in both the numerator and denominator [Figure 1]. Thus, small changes in the repressor scaling factor or cooperativity parameters will have much smaller effects on the overall change in expression. This representation of cooperativity parameters in the model is the standard method of describing interactions in thermodynamic models, thus a resulting low sensitivity may permit more non-physiological parameters to be accepted. Experimental evidence suggests that at least in some cases, however, there should be a high degree of sensitivity to reflect the important protein-protein cooperative interactions on enhancers [29-31].

The mathematical representation of cooperativity in this model is based on the interactions at the transcription factor-DNA binding level, thus cooperativities are represented in the same form in both the numerator and denominator of the expression function [Figure 1]. However, cooperativity between two transcription factors can be represented as a post-binding event, by constructing the model in such a way that all states of the enhancer are considered, then weighted by a cooperativity parameter. In this second case, the model formula would be written exactly the same as Figure 1B except that C1 would not be present in the denominator of the expression. Thus the cooperativity parameters would be present only in the numerator of the expression function, increasing their sensitivity, possibly to levels comparable to quenching parameters.

We noted that quenching parameters' sensitivities varied, with Q6 showing the lowest sensitivity (Figure 3). In the construction of the model, Q6 represents the quenching directed by a lone repressor binding to the 3' end of the regulatory region. There is no biological evidence that suggests a correlation between the particular position and short-range repression effectiveness, but some other model parameter might compensate for changes in Q6. For this reason, and the fact that some quenching parameters are seen in the same number of constructs, but have different sensitivity indices, we turned to another global sensitivity analysis technique to test for possible effects of interactions between parameters.

**Figure 4:**
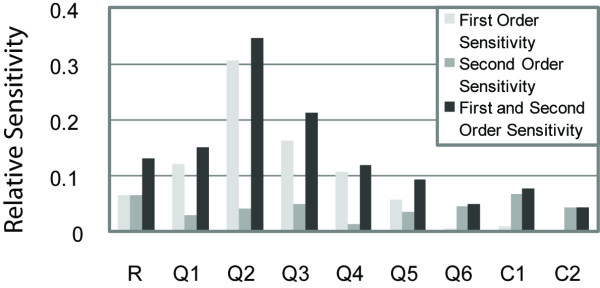
**Sensitivity analysis using the HDMR global sensitivity method on the thermodynamic model of Fakhouri et al., scheme 2**. Shown are first-, second- and the sum of first- and second-order sensitivity indices, which represent the amount of variation in the model output with respect to each parameter individually (light gray bars), each pair of parameters (dark gray bars), and the sum of these two variations (black bars), scaled from 0.0 to 1.0. General sensitivity trends are similar to those observed in Figure 2, although second-order sensitivities are largest for R and C1, implying that they affect the model output through pair-wise interactions with other parameters. Over 95% of the variation in model output was found using only first- and second- order sensitivities (data not shown); therefore the sum of first- and second-order sensitivities shown here approximates the total sensitivity of each parameter.

We tested the HDMR global sensitivity technique, which provides information about inter-parameter sensitivities, on this thermodynamic model. The first and second-order sensitivity results are shown in Figures 4 and S3. Relative overall sensitivities were generally similar to those obtained with eFAST, however, this method also provided insights on possible interactions between quenching, repressor scaling, and cooperativity factors. The five largest second-order sensitivity indices found for one formulation of the model, scheme 2, in descending order, were Q6-C1, Q3-Q5, R-C1, Q2-Q3, and R-C2. Out of the nine schemes tested, C1 was always seen in these top five (Figures 4 and S3). It is interesting that although C1 has a very small first-order sensitivity index, the sum of its second-order sensitivity indices is the largest of any parameter. Biologically, cooperativity between repressors would only have an effect on gene expression by increasing binding strength or quenching efficiency. Hence, C1 only has an impact on the model output when we are also considering the impacts of the repressor scaling factor or the quenching parameters. Mathematically, C1 is always multiplied by a factor containing another parameter; therefore it can easily be compensated for. An increase in C1 with a simultaneous decrease in another parameter would result in no change in the predicted gene expression, hence no change in the RMSE.

HDMR second-order interactions also suggested interactions between quenching parameters. Most often, this was observed between quenching parameters that were present together in more than one construct, such as Q3 and Q5. This effect may be due to compensation in the RMSE from one construct to another. RMSE is calculated by summing up the square errors over all constructs, therefore parameter values that increase the error in one construct, but severely decrease the error in another construct, would leave the RMSE unchanged. Hence, a sensitivity index interpreted as an interaction between two parameters may not be as simple as the two values affecting one another. A high sensitivity index between two quenching parameters may be a result of those two quenching parameters being present together in most constructs. In this case, it would be difficult to remove the dependence of one parameter on another because the parameters appear together in most terms of the RMSE calculation. This effect should be taken into consideration when designing the experiments and developing a model. The best way to avoid this ambiguity would be to design the experiments and the model in unison, to minimize the bias to a certain parameter or group of parameters.

To address this question, we designed new sets of hypothetical genes, created synthetic data sets, and performed global sensitivity analyses (see Additional file 1). The results show that improving construct design by uniformly representing quenching parameters partially equalizes parameter sensitivities, however, quenching parameters still remain more sensitive than repressor-repressor cooperativity parameters (Figure S4, compare to Figure 4). The structure of the mathematical model guarantees this disparity regardless of the experimental design or data.

We also gained insight into the effects the biological data has on parameter sensitivities. Clearly, from a mathematical point of view, the data has some effect on the sensitivity analysis, since the objective function used here is a measure of error. For these new gene constructs, when the data suggests parameter values that lie near the edge of realistic parameter space, we find much higher variation in the sensitivity coefficients of quenching parameters (Figure S4A vs. S4B). This effect is due to random sampling techniques used in global sensitivity methods. When choosing random values in a finite range, there is a higher probability that they lie close to a mean value than a value on either side of the mean. For synthetic data sets created using parameters set at mean values, the error will be minimized, thus decreasing the sensitivity of the parameters. We observe this effect for both the original constructs and the redesigned constructs (Figures S4 and S5). The results validate our hypothesis that the sensitivity coefficients are dependent, at least in part, on the true parameter values, those represented in the experimental data (Figures 3B vs. S5B).

Optimizing the design of constructs to equalize the frequency of parameters does decrease the range of parameter sensitivities, but not completely (Figure S4). Further equalization is possible by ensuring that quenching parameters are represented uniformly in a combinatorial fashion (Figure S6). This analysis on synthetic data underscores the need for global sensitivity analyses on modeling studies with the objective of reducing the error between predictions and experimental data. The analysis should be done both before and after experimental data collection, first to test for uniform sensitivity on synthetic data constructed with mean values; then a second time, to provide a level of confidence in extracted parameter values and their biological implications.

### Global Sensitivity Analysis of the Zinzen et al. Model

**Figure 5:**
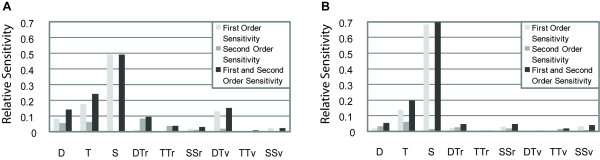
**Sensitivity analysis using the HDMR global sensitivity method on the thermodynamic model of Zinzen et al**. First-, second- and the sum of first- and second-order sensitivity indices (scaled from 0.0 to 1.0), are shown. Despite the focus in the Zinzen et al. study on Dorsal-Twist cooperativity, these parameters are relatively insensitive compared to protein scaling factors. Results from representative enhancer structures: A) set 1 and B) set 2. The model parameters are shown on the horizontal axis. D, T, and S correspond to scaling factors for Dorsal, Twist, and Snail binding sites, respectively. DTr, TTr, and SSr correspond to cooperativity parameters representing Dorsal-Twist, Twist-Twist, and Snail-Snail cooperativities respectively, for the *rho*-like enhancer. Similarly, DTv, TTv, and SSv correspond to cooperativity parameters representing Dorsal-Twist, Twist-Twist, and Snail-Snail cooperativities respectively, for the *vnd*-like enhancer.

The second study that we considered differs considerably from the previous work, in which a limited set of defined gene constructs was quantitatively tested. In Zinzen et al., many conceptual constructs thought to resemble one of two endogenous genes were the subject of analysis. Each virtual construct is a list of binding sites for activator and repressor proteins, with no information about the arrangement or spacing of the sites. The only difference between constructs is the number of transcription factor binding sites. The structures of the constructs simulate *rho *or *vnd*, two genes with slightly differing expression patterns in the Drosophila embryo. The derived parameters were used to infer possible regulatory relationships explaining the design of enhancers controlling *rho *and *vnd*. We applied the HDMR approach to their model to obtain information on sensitivities revealed by this method. Zinzen et al. used the Pearson correlation coefficient to determine the quality of fit, therefore, for the sensitivity analysis of this model, we used this correlation coefficient as the objective function. In their study, twelve parameter combinations and eight unique enhancer structures were identified as most informative, after fitting all possible forms of their model to expression data [2]. We used these eight enhancer structures for our global sensitivity analysis. Each enhancer set is associated with 9 parameters: 3 scaling factors reflecting transcription factor activity, and 3 different cooperativity parameters for each of the *rho*-like and *vnd*-like enhancer structures. The first and second-order parameter sensitivity results for two representative pairs of enhancer structures are shown in Figure 5 (additional results in Figure S7).

Strikingly, transcription factor scaling factors are far more sensitive than the cooperativity parameters for almost every enhancer structure tested (Figures 5 and S7). The overall low sensitivities in cooperativity parameters do not necessarily contradict the conclusions made by the authors that different cooperativity parameters distinguish the expression patterns of *rho *and *vnd*, but they do draw into question how robust these conclusions can be [2]. Low sensitivity values do not mean that the parameters are of low importance, but rather indicate that changes in these parameter values do not have large effects on the output. Therefore, other sets of parameter values, with different relative importance of cooperativity parameters, may also allow the model to distinguish the expression patterns of *rho *and *vnd*. The study claimed that *rho*-like elements require 5-10 fold-higher Dorsal-Twist cooperativity, higher Twist-Twist cooperativity and lower Sna-Sna cooperativity than do *vnd*-like elements [2]. We explored the correlation in other regions of parameter space to determine how strict these relationships are and how much changes in these relationships would affect the model's correlation with experimental data.

By investigating the parameter values listed in their study, a reasonable range for the parameter values was deduced [2]. The scaling factors were taken in the range [10^8^,10^10^] and cooperativity values were taken in the range [1,100]. For each enhancer structure, we chose 100,000 sets of parameter values at random and calculated the Pearson correlation coefficients. We sorted the Pearson correlation coefficient values and selected for further analysis all parameter sets that resulted in values greater than 0.981, a threshold chosen based on the lowest value that appeared in Table S2 (log[correlation coefficient] = 1.72) [2]. We obtained between 105 to 174 acceptable parameter sets from each of the original 100,000 parameter sets for each enhancer structure. The results are shown in Table 1, which illustrates how often the relationships between cooperativity parameters in elements chosen to resemble *rho *and *vnd *were exactly as predicted by Zinzen et al., and how often some or all of these relationships could be changed, yet still recover a satisfactory correlation (greater than 0.981). We carried out this test a second time, again testing 100,000 different random parameter sets for each construct and obtained essentially identical results (data not shown). The relationships that were said to be of greatest importance in Zinzen et al. appear in a plurality of parameter sets in almost every scheme, but other relationships between the cooperativity parameters also result in a very high correlation. The relationships predicted in Zinzen et al. hold for less than 40% (between 13 and 59 of between 105 and 174) of the parameter sets that show satisfactory correlation (> 0.981). As modeled in that study, the relationships may not represent biological features that reflect a compelling best fit with the experimental data; rather, the low sensitivities of these particular parameters may allow one to accept a local best fit. The biological conclusions, that *rho *and *vnd *expression patterns differ based on Twist-Dorsal cooperativity, should therefore be interpreted with caution.

**Table 1:** Parameter relationships resulting in correlations as good as those found in Zinzen et al. from 100,000 tested parameters

Set(s)	all correct	DT wrong	TT wrong	SS wrong	DT and TT wrong	DT and SS wrong	TT and SS wrong	all wrong	total
1	57 (39%)	6 (4%)	43 (29%)	20 (14%)	0 (0%)	2 (1%)	18 (12%)	0 (0%)	146
2	49 (28%)	32 (18%)	45 (26%)	16 (9%)	8 (5%)	9 (5%)	13 (7%)	2 (1%)	174
3 and 9	59 (38%)	10 (6%)	47 (31%)	13 (8%)	1 (1%)	5 (3%)	19 (12%)	0 (0%)	154
4 and 7	48 (39%)	1 (1%)	44 (36%)	17 (14%)	0 (0%)	0 (0%)	13 (11%)	0 (0%)	123
5, 6, and 8	37 (35%)	2 (2%)	34 (32%)	17 (16%)	0 (0%)	0 (0%)	15 (14%)	0 (0%)	105
10	13 (11%)	4 (3%)	17 (15%)	29 (25%)	8 (7%)	12 (10%)	21 (18%)	13 (11%)	117
11	36 (24%)	1 (1%)	30 (20%)	37 (25%)	0 (0%)	8 (5%)	36 (24%)	0 (0%)	148
12	36 (28%)	3 (2%)	16 (13%)	38 (30%)	0 (0%)	3 (2%)	32 (25%)	0 (0%)	128

From those parameter sets with a Pearson correlation coefficient greater than 0.981 (last column) the relationships highlighted by Zinzen et al. are observed in the largest proportion, although still less than 40% of the time. The first column lists the sets of enhancer structures used to calculate the Pearson correlation coefficient for each random set of parameter values. The numbering of the enhancer structure sets is consistent with Table S2 of the Zinzen et al. study. Columns 2-9 list the number of parameter sets that fall into each relationship category, compared to those relationships stated in Zinzen et al., DTr > DTv, TTr > TTv, and SSr < SSv, and the corresponding percentage, rounded to the nearest percent, of the parameter sets tested that fall into each category.

Table 1 also indicates how sensitivity analysis can guide model selection. The insensitivity of cooperativity parameters led us to test a large set of different parameter values, which revealed that multiple parameter sets with different biological interpretations produced satisfactory correlations between the experimental data and the model output. This modeling weakness may reflect the reliance on only one experimental data set to fit parameter values. In addition, information about positions of binding sites in enhancers was not considered, and somewhat arbitrary assumptions about which proteins are required for activity were made. Any of these factors may have led to insensitivity in the cooperativity parameters, and should be addressed before trying to improve upon the model or its biological interpretations.

## Discussion

Thermodynamic models represent the most powerful approach to quantitative understanding of gene transcription because of their use of DNA sequence information that can capture subtle differences in enhancer architecture. As with other models, this approach involves many unknown parameters and interactions, however, a rigorous sensitivity analysis has not been applied to recent studies in this area. We show here, through local and global sensitivity analyses, that there is a wide range in sensitivities for parameters in these models. In some cases, this is due to the biology of the system, or the experimental data used to calculate the error, while in other cases it may simply be an artifact of the mathematical formulation (Figure 1), and the underlying assumptions that have gone into this model.

Our analysis also uncovered previously undescribed interactions between parameters that may reflect true biological interactions, and at the same time found very low sensitivities in parameters that were previously thought to be most informative in explaining the overall architecture of distinct enhancers. We used the insights drawn from sensitivity analysis to redesign experiments, and discover new parameter values that are well correlated with experimental data. Clearly, standard parameter estimation techniques used alone, in the absence of sensitivity analysis, cannot fully show the importance of possible biologically-driven or model-driven effects.

To improve upon thermodynamic modeling attempts to unlock key aspects of cis-regulatory grammar, we should recognize a need for sensitivity analysis and a thorough exploration of parameter space. With this analysis, new iterative parameter estimation and model selection techniques can be designed and implemented [17,32]. Analysis of the effects parameters have on the model output, within their realistic ranges, can also help biologists to design experiments. As we demonstrated with our synthetic data sets, a tight coordination between experimental design and model formulation is required to maximize the effectiveness of these modeling studies. A conscious effort can be made to collect data evenly and models can be redesigned to make sure that model sensitivity is similar for various parameters. Thermodynamic models, as other models, benefit by such sensitivity analysis, providing important context in which to place biological interpretations suggested by the model.

## Conclusions

Mathematical models of transcription are becoming increasingly widely applied, but analysis of the sensitivity of fractional occupancy (thermodynamic) models has lagged. We have examined two recent thermodynamic modeling studies of transcriptional regulation and shown how parameter sensitivities vary widely. From this examination, we have identified both biological and mathematical bases to differential sensitivity.

In our sensitivity analysis of the Fakhouri et al. model, we first observed that quenching parameters were found to be more sensitive than other model parameters and cooperativity parameters to be least sensitive. Through careful inspection of the model formulation, we concluded that this differential sensitivity was heavily dependent on the mathematical form of the model. We also noted that parameters that were represented in a larger portion of the data set were more sensitive than others. This effect can neither be attributed solely to the mathematical form of the model nor solely to the biology of the system; it is a combination of the two, which emphasizes the importance of designing the model and the experiments in unison. We created synthetic data sets and conducted global sensitivity analyses using these sets as our experimental data, and show that not only an evenly designed data set is important, but the parameter values as represented in the biological data also have a large impact on parameter sensitivities. Thus, sensitivity analysis is not only useful in dissecting the mathematical model formulation, but also in investigating the real parameter space and the impact the data set will have on the model's sensitivity to certain parameters.

Our examination of the Zinzen et al. model produced similar results, in which cooperativity parameters were the least sensitive of all parameters in almost every formulation of their model. We investigated these cooperativity parameters and found that this low sensitivity allowed for a wide array of acceptable cooperativity values, all of which showed high correlation with experimental data. Importantly, these alternative cooperativity values lead to very different biological interpretations of the system under investigation. Without thorough sensitivity analysis and parameter space exploration, parameter values extracted using thermodynamic models may be easily misinterpreted.

Our study underscores the importance of carrying out sensitivity analysis to identify model- and data-driven influences on parameters, and to improve the construction and interpretation of transcriptional models based on thermodynamic approaches.

## Authors' contributions

JMD carried out the global sensitivity analysis, the creation of the synthetic data sets, and the data analysis, participated in the design of the study, and drafted the manuscript. XL carried out the local sensitivity analysis, helped to draft the manuscript and created the figures. DNA participated in the design of the study and writing the manuscript. AA conceived of the study, participated in the data analysis, and helped in writing the manuscript. All authors have read and approved the final manuscript.

## Supplementary Material

Additional file 1 **Dresch et al. 2010**. This file includes all supporting text, figures, and tables in pdf format. Click here for file
