# Supplementary material for: Thermodynamic modeling of transcription: sensitivity analysis differentiates biological mechanism from mathematical model-induced effects
Source: BMC Syst Biol. 2010 Oct 24;4:142. doi: 10.1186/1752-0509-4-142 (PMC2987881; doi:10.1186/1752-0509-4-142)

Supporting Information

Error Calculation: RMSE and Pearson Correlation Coefficient

The root mean square error (RMSE), as implemented in the Fakhouri et al. study, was calculated using Giant protein concentrations as model inputs and the corresponding *lacZ* mRNA concentrations as outputs [1]. Data points were taken at 20 different Giant concentration levels, uniformly ranging from 0 to 1. Each term in the RMSE was weighted by the number of actual data points over which each *lacZ* concentration was averaged. In our sensitivity analysis, this weighted RMSE was used as the objective function.

The Pearson correlation coefficient, as implemented in the Zinzen et al. study, was calculated using Dorsal, Twist, and Snail protein concentrations as model inputs and the corresponding *rho* and *vnd* mRNA concentrations as outputs, which can be found at DVEx database (http://www.dvex.org) [2].

Enhancer-like structures used to calculate sensitivities of the Zinzen et al. model

For the results shown in Figure 3, we used two representative pairs of enhancer structures proposed by Zinzen et al.. The remaining structures were analyzed as shown in Figure S7. Each representative pair of enhancer structures is defined by the number of modules and binding sites in each of the representative enhancers. An enhancer with more than one module (Md > 1) is assumed to have identical, independent modules contributing to the expression of that gene. D, T/S, T, and S represent the number of binding sites for Dorsal, Twist/Snail, Twist, and Snail, respectively, in each module. A T/S binding site differs from other binding sites in that it can be occupied by either the Twist or Snail protein. The first set of representative enhancers has, for the *rho*-like

enhancer: Md = 1, D = 2, T/S = 2, T = 1, S = 2 and for the *vnd*-like enhancer: Md = 2, D = 2, T/S = 1, T = 1, S = 2 [2]. The second has, for the *rho*-like enhancer: Md = 1, D = 2, T/S = 1, T = 2, S = 2 and for the *vnd*-like enhancer: Md = 2, D = 2, T/S = 0, T = 2, S = 2 [2]. For the description of the remaining representative enhancers, refer to [2].

eFAST Implementation

The eFAST algorithm differs from the original FAST algorithm only in the calculation of total order sensitivity indices [3-4]. For the model given by the function $f(x)$, where x is the vector of parameter values, the first order sensitivity index for each parameter, x_i, where $i = 1,...,n$ and n is the number of parameters, is calculated in the following way:

1. A sinusoidal function (search curve) $x_i = g_i(j;s)$, is defined with $j = 1,...,N_S$, where j values correspond to integer frequencies and N_S corresponds to the sample size. One example of such a search curve is [4]:

$$g_i(j;s) = \frac{1}{2} + \frac{1}{\pi}\arcsin(\sin js)$$

For a detailed description of how search curves are specified, see [3].

2. Fourier coefficients are then calculated for the search curve at each frequency, j, using the following formulas:

$$A_j = \int_{-\pi}^{\pi} f(s)\cos(js)ds$$

$$B_j = \int_{-\pi}^{\pi} f(s)\sin(js)ds$$

where $f(s) = \sum_{j=-\infty}^{\infty}\left(A_j \cos js + B_j \sin js\right) = f\left(g_1(j;s),...,g_n(j;s)\right)$

3. The variance at each frequency, j, and the total variance are then calculated using these Fourier coefficients in the following way:

$$\sigma_{i,j}^2 = 2\left(A_j^2 + B_j^2\right)$$

$$\sigma_{i,\,total}^2 = \sum_{j=1}^{N_S} \sigma_{i,j}^2$$

4. The first order sensitivity index for parameter i is then calculated using the variance at the fundamental frequency, ω, as a fraction of the total variance in the following way:

$$S_i = \frac{\sigma_{i,\,\omega}^2}{\sigma_{i,\,total}^2}$$

The fundamental frequencies are chosen so that each parameter with a strong influence on the model output will produce oscillations of high amplitude at the fundamental frequency [4]. Additionally, the fundamental frequencies, ω's, are bounded by the relation:

$$N_S = 2M \max\{\omega\} + 1$$

where M is the interference factor and is typically set to be 4 or higher [4].

For more details on the recursive algorithm used to calculate these fundamental frequencies see [4]. There is an additional parameter , N_R, which corresponds to the resampling size. Resamplings simply introduce phase shifts to the search curve, increasing the number of total samples in parameter space. When $N_R > 1$, S_i is calculated using the mean of the variances.

The calculation of the total order sensitivity index of a parameter is then easily computed by combining other parameters into a single group (with one fundamental frequency) and calculating the first order sensitivity of this complementary group of

parameters, S_{Ci}, and removing this proportion of variance from the total proportion of variance. Thus, the total order sensitivity index of the parameter i is computed as:

$S_{T_i} = 1 - S_{Ci}$

We used the eFAST algorithm, as written by Marino et al. [3]. Modifications to this algorithm involved removing time-dependence, including our objective function, and setting baseline parameter values based on those published in [1]. eFAST parameters were determined from the formulation recommended in [4]. For scheme 2, the parameters were set at k = 10, $N_S = 73$, $N_R = 100$, and M = 4.

HDMR Implementation

The theoretical foundations of the HDMR algorithm are almost identical to that of eFAST. Both algorithms originate from ideas of Sobol' and Cukier of decomposing the variance into its contribution from each input parameter. The HDMR algorithm we employed uses a different method of searching parameter space than that of eFAST. Instead of defining search curves as 1-dimensional sinusoidal functions, the algorithm begins with a quasi-random sampling of parameter space obtained using Sobol' sequences. Before constructing the quasi-random sampling, the first step is to rescale the parameter ranges to [0,1]. This allows us to think of the model function, $f(x)$, on the domain defined by the unit hypercube, $K^n = \{(x_1, x_2, ..., x_n) | 0 \le x_i \le 1, \; i = 1,2,...,n\}$.

As stated in the main text, the HDMR algorithm, or ANOVA, decomposes a function into a summation of terms of increasing dimensionality [5]:

$$f(x) = f_0 + \sum_{i=1}^{n} f_i(x_i) + \sum_{1 \le i < j \le n} f_{ij}(x_i, x_j) + ... + f_{12...n}(x_1, x_2, ..., x_n)$$

This decomposition always contains a finite number of terms and is exact. In this study, we have approximated the model function up to second order, thus we use the approximation:

$$f(x) \approx f_0 + \sum_{i=1}^{n} f_i(x_i) + \sum_{1 \le i < j \le n} f_{ij}(x_i, x_j)$$

When a quasi-random sampling of parameter space is taken, a set of *N* parameter sets are obtained. By using these parameter sets as inputs to the model, *N* output values, $f(x^k)$, $k = 1,...,N$ are obtained. The first term in the decomposition of *f*, the main effect term, f_0 , is simply calculated as the overall mean of these *N* outputs:

$$f_0 = \frac{1}{N} \sum_{k=1}^{N} f(x^k)$$

The higher order terms in the expansion are then approximated by orthonormal polynomials, $\varphi_1, \varphi_2, \varphi_3, ...$, with $\deg(\varphi_r) = r$. In this study, we used orthonormal polynomials up to degree 5 for first order approximations and up to degree 3 for second order approximations. Thus, the approximations were the following:

$$f_i(x_i) \approx \sum_{r=1}^{5} \alpha_r^i \varphi_r(x_i)$$

$$f_{ij}(x_i, x_j) \approx \sum_{r=1}^{2} \sum_{\substack{l=1 \\ l+r \le 3}}^{2} \beta_{rl}^{ij} \varphi_r(x_i) \varphi_l(x_j)$$

where α_r^i and β_{rl}^{ij} are constant coefficients determined by minimizing the square of the difference between the function and the approximation:

$$f(x) \approx \frac{1}{N} \sum_{k=1}^{N} f(x^k) + \sum_{i=1}^{n} \sum_{r=1}^{5} \alpha_r^i \varphi_r(x_i) + \sum_{1 \le i < j \le n} \sum_{r=1}^{2} \sum_{\substack{l=1 \\ l+r \le 3}}^{2} \beta_{rl}^{ij} \varphi_r(x_i) \varphi_l(x_j)$$

using the output values obtained from the random sampling.

We used the HDMR algorithm, as written by Ziehn and Tomlin [5]. This algorithm required a matrix of input values (9x1024) corresponding to the 9 different parameter values used for 1024 different runs of the model and a vector of output values (1024 RMSEs or 1024 Pearson correlation coefficients obtained). The input values were created pseudo-randomly, using Sobol' sequences, and the HDMR-GUI was run under its default settings.

Optimized Gene Construct Design and Synthetic Data

To test the effect of experimental design on the sensitivity analysis, we redesigned eleven gene constructs so that each quenching parameter is represented in exactly 3 constructs (Table S1). We then created a synthetic data set of Giant protein and corresponding *lacZ* mRNA concentrations, using the model formula and the parameter values estimated in the Fakhouri et al. study. The eFAST and HDMR analyses gave comparable results, and showed a decrease in the variation of the parameter sensitivities when compared to Figure 4, although not a perfectly even distribution (HDMR results in Figure S4A, eFAST results not shown). We hypothesized that this was either due to the parameter values used to create our synthetic data set, or to uneven parameter interactions in the redesigned constructs. To test our first hypothesis, we created a new synthetic data set for these eleven gene constructs using the mean values in each parameter range (0.5 for quenching parameters, 50 for scaling factor and cooperativity parameters). The results show only a slight decrease in the variation of sensitivities (Figure S4B). This decrease can be seen by comparing parameters Q2 and Q5 with Q6 (Figure S4A vs. S4B; Q2, Q5, and Q6 have been changed from 0.31, 0.20, and 0.62 respectively to 0.50). To further investigate this point, we created a new synthetic data set using the mean value in

each parameter range for the twelve original gene constructs used by Fakhouri et al. We observed a slight decrease in variation of sensitivities, and a stronger correlation between the number of constructs in which a parameter is represented and the sensitivity of the parameters (Figure S5 vs. 3B). In neither of the above cases were the constructs perfectly combinatorial. Although in the new design each quenching parameter is represented in the same number of constructs, they are not represented in the same context. To test the "best" combinatorial enhancer design, we considered only four quenching parameters (three representing quenching at various distances 5' of the activator binding sites and one representing quenching from the 3' direction) and designed a perfect combinatorial set of fifteen enhancers, including all possible combinations of these four parameters (Table S2). From this set of fifteen enhancers, we then chose two separate subsets to test: the first subset (Constructs 1,2,3,4,11,12,13,14,15) was perfectly even in the sense that each quenching parameter was represented in exactly the same number of constructs and with the same number of other quenching parameters, and the second subset (Constructs 1,2,5,6,7,8,11,12,13) was uneven, with Q1 represented 7 times, Q2 5 times, Q3 4 times, and Q4 3 times. We then created synthetic data sets using the mean parameter values. We observed that with a perfectly even experimental design and mean parameter values, minimal variation is seen in the sensitivities of quenching parameters (Figure S6A). This is not the case with an uneven experimental design even with mean parameter values (Figure S6B). Thus, our results have shown that parameter sensitivities have a clear dependence on both the experimental design and true biological parameter values (Figures S4-S6).

References

1. Fakhouri WD, Ay A, Sayal R, Dresch J, Dayringer E, Arnosti DN: **Deciphering a transcriptional regulatory code: modeling short-range repression in the Drosophila embryo.** *Mol Syst Biol* 2010, **6**: 341.
2. Zinzen RP, Senger K, Levine M, Papatsenko D: **Computational models for neurogenic gene expression in the Drosophila embryo**. *Curr Biol* 2006, **16**: 1358–1365
3. Marino S, Hogue IB, Ray CJ, Kirschner DE: **A methodology for performing global uncertainty and sensitivity analysis in systems biology**. *J Theor Biol* 2008, **254:** 178-196.
4. Saltelli A, Tarantola S, Chan KPS: **A quantitative model-independent method for global sensitivity analysis of model output**. *Technometrics* 1999, **41:** 39-56.
5. Ziehn T, Tomlin AS: **A global sensitivity study of sulfur chemistry in a premixed methane flame model using HDMR**. *Int J Chem Kinet* 2008, **40:** 742-753.

Figures

Figure S1 - Sensitivity analysis using the global-local method on the thermodynamic model of Fakhouri et al., schemes 1 and 3-9.

As seen in Figure 1, for scheme 2, quenching parameters (Q) are generally more sensitive than repressor scaling factors (R), which in turn are more sensitive than cooperativity parameters (C), although there is some variation from scheme to scheme. The model

parameters are shown on the horizontal axis and global-local sensitivity contributions are plotted on the vertical axis.

Figure S2 - Sensitivity analysis using the eFAST global sensitivity method on the thermodynamic model of Fakhouri et al., schemes 1 and 3-9, reveals inter-parameter interactions.

As observed in Figure 2, for scheme 2, quenching parameters are generally most sensitive, cooperativity parameters are least sensitive, and the more constructs a parameter is represented in, the more sensitive that parameter. An exception to this is seen in F), scheme 7, where there does not appear to be any correlation between a parameter's sensitivity coefficient and the number of constructs it is represented in. Parameters of low sensitivity exhibit larger relative proportions of non-first order effects. The number of constructs that the parameter is represented in is shown in brackets below the parameter. Corresponding first- and total-order sensitivity indices, which represent the amount of variation in model output with respect to each parameter individually and in conjunction with all other parameters, are shown.

Figure S3 - Sensitivity analysis using the HDMR global sensitivity method on the thermodynamic model of Fakhouri et al., schemes 1 and 3-9.

General sensitivity trends are similar to those observed in Figures 2 and 3. The model parameters are shown on the horizontal axis. Corresponding sensitivity values are shown for each parameter, as calculated by the HDMR algorithm. The sum of second-order sensitivities represents the amount of variation accounted for by pair-wise interactions with other model parameters.

Figure S4 - Sensitivity analysis using the HDMR global sensitivity method on 11 redesigned gene constructs containing equal numbers of quenching parameters (see Table S1).

Synthetic data created using the Fakhouri et al. model on these 11 redesigned gene constructs shows a decrease in variation of sensitivities between quenching parameters compared to Figure 3. In A, the synthetic data was created using the parameter values that were found in the Fakhouri et. al study. In B, the synthetic data was created using mean parameter values (0.5 for quenching parameters, 50 for scaling factor and cooperativity parameters). A decrease in variation of sensitivities for quenching parameters is also observed from A to B (the sum of first and second order sensitivities has a range of 0.10 to 0.28, with a mean of 0.17 and a standard deviation of 0.07 in A and a range of 0.16 to 0.31, with a mean of 0.20 and a standard deviation of 0.06), indicating that the sensitivity analysis is dependent on the true parameter values represented in the biological data.

Figure S5 - Values of biological data can affect sensitivity analysis.

The eFAST global sensitivity method was applied to the thermodynamic model of Fakhouri et al., scheme 2, using synthetic data with mean values. We observe a decrease in variation of sensitivities between quenching parameters, when compared to Figure 2. A) Sensitivity indices represent the amount of variation in the RMSE, calculated using all 12 constructs and synthetic data created using mean parameter values (0.5 for quenching parameters, 50 for scaling factor and cooperativity parameters). B) Effect of frequency with which a quenching parameter is represented in the twelve constructs on sensitivity. The number of constructs that the parameter is represented in is shown along the

horizontal axis. Corresponding first- and total-order sensitivity indices are shown for each quenching parameter, as calculated by the eFAST algorithm. Note that there are two quenching parameters (Q4 and Q5) represented in two constructs, and two quenching parameters (Q1 and Q3) represented in five constructs. Thus at these values there are four data points, two for each quenching parameter. The lines illustrate linear fits to each data set, first- and total-order sensitivity indices. In general, the more constructs a parameter is represented in, the more sensitive that parameter. When compared to Figure 2B, a more pronounced correlation between a parameter's total-order sensitivity coefficient and the number of gene constructs that parameter is represented in is observed here, underscoring the need for sensitivity analysis in experimental design.

Figure S6 - Effect of combinations of four quenching parameters on sensitivity analysis using the HDMR global sensitivity method on redesigned gene constructs (see Table S2).
Compared to an even design, an increase in variation of sensitivities between quenching parameters is seen when the data used comes from an uneven construct design (compare A to B). The synthetic data was created using the 9 constructs in which the quenching parameters were evenly distributed (A) and not evenly distributed (B). Mean parameter values (0.5 for quenching parameters, 50 for scaling factor and cooperativity parameters) were used in both cases.

Figure S7 - Sensitivity analysis using the HDMR global sensitivity method on the thermodynamic model of Zinzen et al., enhancer structure sets 3-8.
As observed in Figure 4, for sets 1 and 2, protein scaling factors are considerably more sensitive than the cooperativity parameters. The model parameters are shown on the

horizontal axis. D, T, and S correspond to scaling factors for Dorsal, Twist, and Snail binding sites, respectively. DTr, TTr, and SSr correspond to cooperativity parameters representing Dorsal-Twist, Twist-Twist, and Snail-Snail cooperativities respectively, for the *rho*-like enhancer. Similarly, DTv, TTv, and SSv correspond to cooperativity parameters representing Dorsal-Twist, Twist-Twist, and Snail-Snail cooperativities respectively, for the *vnd*-like enhancer.

Tables

Table S1. Redesigned gene constructs to provide uniformity in the number of quenching terms.

Gene Construct	R	Q1	Q2	Q3	Q4	Q5	Q6	C1	C2
1. 1Gt_2Tw2Dl	x	x							
2. 2Tw2Dl_1Gt	x						x		
3. 1Gt_25_2Tw2Dl	x		x						
4. 1Gt_32_1Gt_2Tw2Dl	x	x		x					x
5. 2Gt_50_2Tw2Dl	x			x		x		x	
6. 1Gt_32_1Gt_35_2Tw2Dl	x		x			x			x
7. 2Gt_60_2Tw2Dl	x				x			x	
8. 2Gt_35_2Tw2Dl_1Gt	x		x		x		x	x	
9. 3Gt_50_2Tw2Dl	x			x		x		x	x
10. 1Gt_2Tw2Dl_1Gt	x	x					x		
11. 3Gt_60_2Tw2Dl	x				x			x	x

In the first column the number and name given to each gene construct is listed. “Gt”, “Tw”, and “Dl” represents a Giant, Twist, and Dorsal binding sites, respectively. Numbers in between binding sites represent the number of base pairs present in the DNA spacer between binding sites. Columns 2-10 correspond to the parameters as they defined in scheme 2 of the Fakhouri et al. study. An ‘x’ corresponds to the parameter being present in the model formulation of that construct and no ‘x’ corresponds to the

parameter being absent in the model formulation of that construct. All quenching terms are found in exactly three constructs.

Table S2. Gene constructs that feature all possible combinations of four quenching parameters.

Gene Construct	R	Q1	Q2	Q3	Q4	C1	C2
1. 1Gt_2Tw2Dl	x	x					
2. 1Gt_15_2Tw2Dl	x		x				
3. 1Gt_30_2Tw2Dl	x			x			
4. 2Tw2Dl_1Gt	x				x		
5. 2Gt_2Tw2Dl	x	x	x			x	
6. 1Gt_15_1Gt_2Tw2Dl	x	x		x			x
7. 1Gt_2Tw2Dl_1Gt	x	x			x		
8. 2Gt_15_2Tw2Dl	x		x	x		x	
9. 1Gt_15_2Tw2Dl_1Gt	x		x		x		
10. 1Gt_30_2Tw2Dl_1Gt	x			x	x		
11. 3Gt_2Tw2Dl	x	x	x	x		x	x
12. 2Gt_2Tw2Dl_1Gt	x	x	x		x	x	
13. 1Gt_15_1Gt_2Tw2Dl_1Gt	x	x		x	x		x
14. 2Gt_15_2Tw2Dl_1Gt	x		x	x	x	x	
15. 3Gt_2Tw2Dl_1Gt	x	x	x	x	x	x	x

In the first column the number and name given to each gene construct is listed. Columns 2-8 correspond to the newly defined parameters. Q1-Q3 represent quenching at increasing distances 5’ of the activator binding sites and Q4 represents quenching from the 3’ direction. The repressor scaling factor and cooperativity parameters are defined in the same way in which they are defined in scheme 2 of the Fakhouri et al. study. An ‘x’ corresponds to the parameter being present in the model formulation of that construct and no ‘x’ corresponds to the parameter being absent in the model formulation of that construct.

Figure S1.

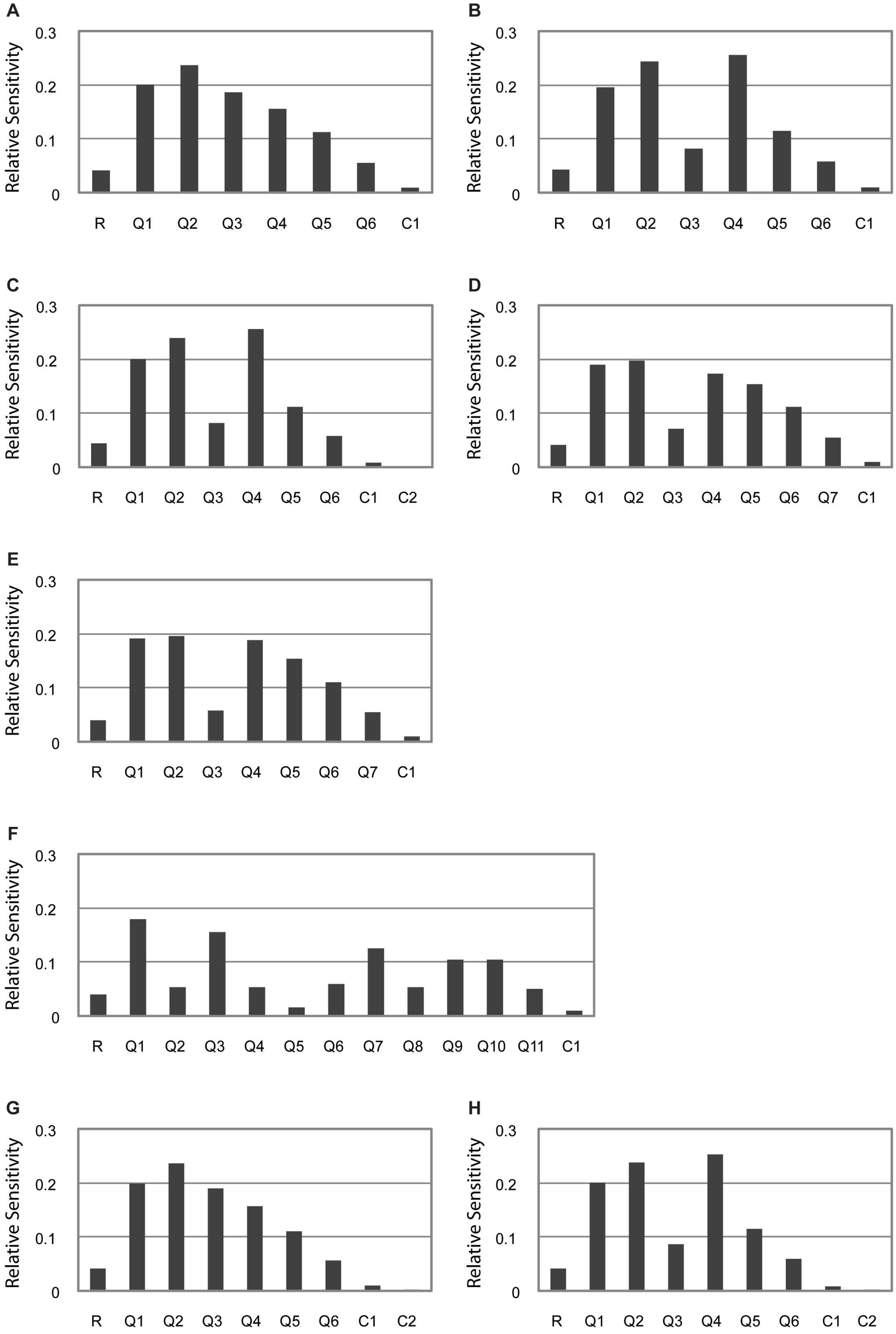
A
Relative Sensitivity
0.3
0.2
0.1
0
R Q1 Q2 Q3 Q4 Q5 Q6 C1
B
Relative Sensitivity
0.3
0.2
0.1
0
R Q1 Q2 Q3 Q4 Q5 Q6 C1
C
Relative Sensitivity
0.3
0.2
0.1
0
R Q1 Q2 Q3 Q4 Q5 Q6 C1 C2
D
Relative Sensitivity
0.3
0.2
0.1
0
R Q1 Q2 Q3 Q4 Q5 Q6 Q7 C1
E
Relative Sensitivity
0.3
0.2
0.1
0
R Q1 Q2 Q3 Q4 Q5 Q6 Q7 C1
F
Relative Sensitivity
0.3
0.2
0.1
0
R Q1 Q2 Q3 Q4 Q5 Q6 Q7 Q8 Q9 Q10 Q11 C1
G
Relative Sensitivity
0.3
0.2
0.1
0
R Q1 Q2 Q3 Q4 Q5 Q6 C1 C2
H
Relative Sensitivity
0.3
0.2
0.1
0
R Q1 Q2 Q3 Q4 Q5 Q6 C1 C2

Figure S2.

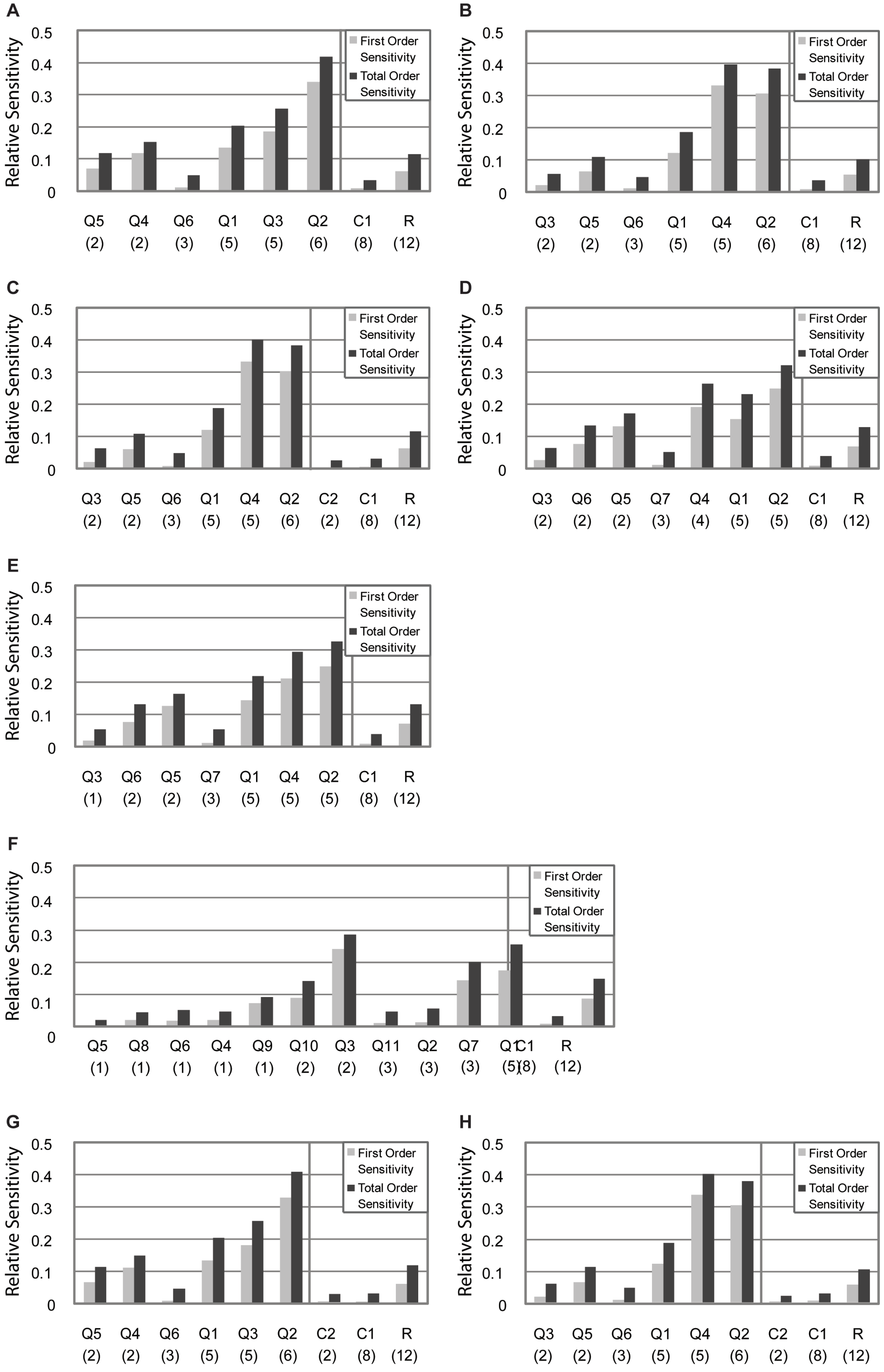
A
Relative Sensitivity
0.5 0.4 0.3 0.2 0.1 0
First Order Sensitivity
Total Order Sensitivity
Q5 (2) Q4 (2) Q6 (3) Q1 (5) Q3 (5) Q2 (6) C1 (8) R (12)
B
Relative Sensitivity
0.5 0.4 0.3 0.2 0.1 0
First Order Sensitivity
Total Order Sensitivity
Q3 (2) Q5 (2) Q6 (3) Q1 (5) Q4 (5) Q2 (6) C1 (8) R (12)
C
Relative Sensitivity
0.5 0.4 0.3 0.2 0.1 0
First Order Sensitivity
Total Order Sensitivity
Q3 (2) Q5 (2) Q6 (3) Q1 (5) Q4 (5) Q2 (6) C2 (2) C1 (8) R (12)
D
Relative Sensitivity
0.5 0.4 0.3 0.2 0.1 0
First Order Sensitivity
Total Order Sensitivity
Q3 (2) Q6 (2) Q5 (2) Q7 (3) Q4 (4) Q1 (5) Q2 (5) C1 (8) R (12)
E
Relative Sensitivity
0.5 0.4 0.3 0.2 0.1 0
First Order Sensitivity
Total Order Sensitivity
Q3 (1) Q6 (2) Q5 (2) Q7 (3) Q1 (5) Q4 (5) Q2 (5) C1 (8) R (12)
F
Relative Sensitivity
0.5 0.4 0.3 0.2 0.1 0
First Order Sensitivity
Total Order Sensitivity
Q5 (1) Q8 (1) Q6 (1) Q4 (1) Q9 (1) Q10 (2) Q3 (2) Q11 (3) Q2 (3) Q7 (3) Q1 (5) C1 (8) R (12)
G
Relative Sensitivity
0.5 0.4 0.3 0.2 0.1 0
First Order Sensitivity
Total Order Sensitivity
Q5 (2) Q4 (2) Q6 (3) Q1 (5) Q3 (5) Q2 (6) C2 (2) C1 (8) R (12)
H
Relative Sensitivity
0.5 0.4 0.3 0.2 0.1 0
First Order Sensitivity
Total Order Sensitivity
Q3 (2) Q5 (2) Q6 (3) Q1 (5) Q4 (5) Q2 (6) C2 (2) C1 (8) R (12)

Figure S3.

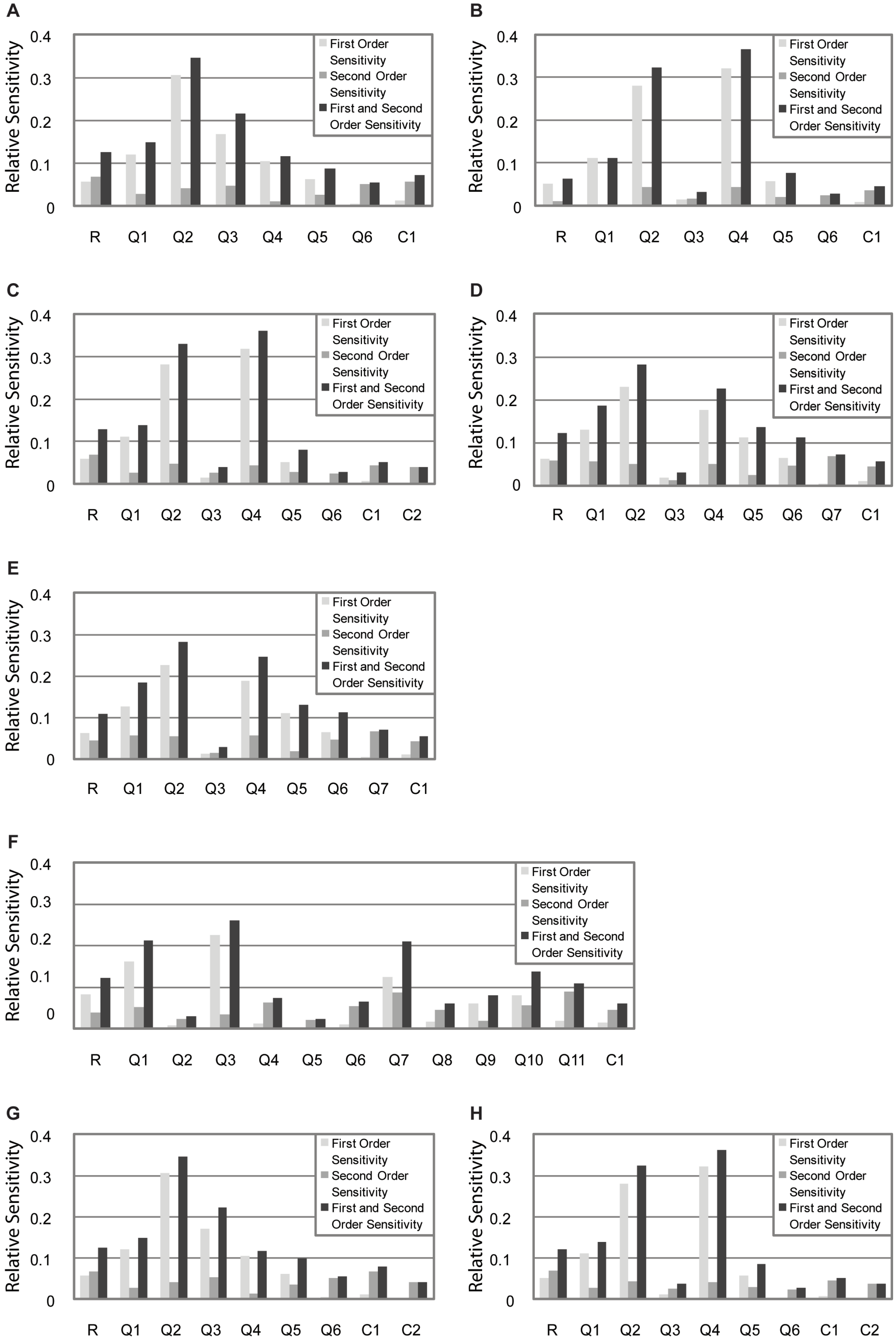

A
B
C
D
E
F
G
H
Relative Sensitivity
0
0.1
0.2
0.3
0.4
First Order Sensitivity
Second Order Sensitivity
First and Second Order Sensitivity
R
Q1
Q2
Q3
Q4
Q5
Q6
Q7
Q8
Q9
Q10
Q11
C1
C2

Figure S4.

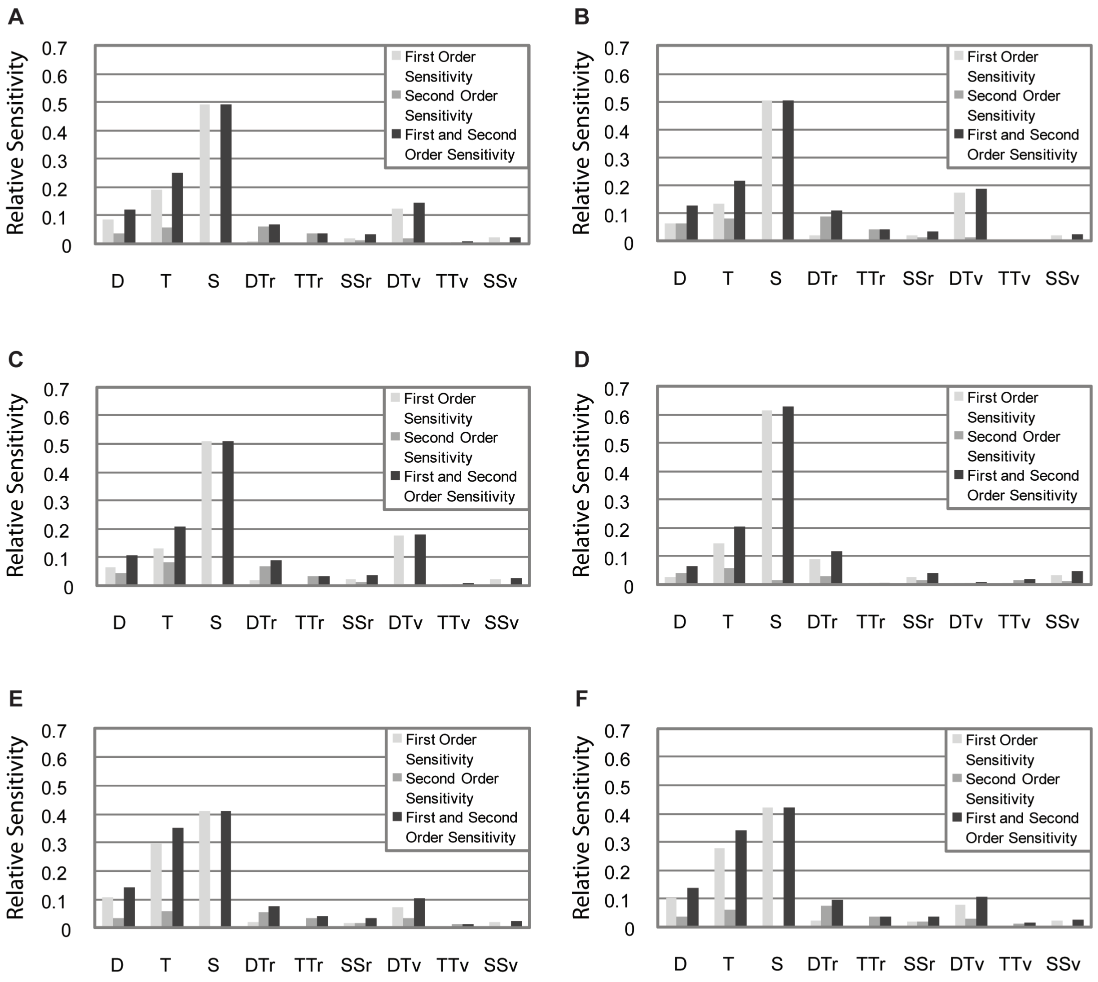

Figure S5.

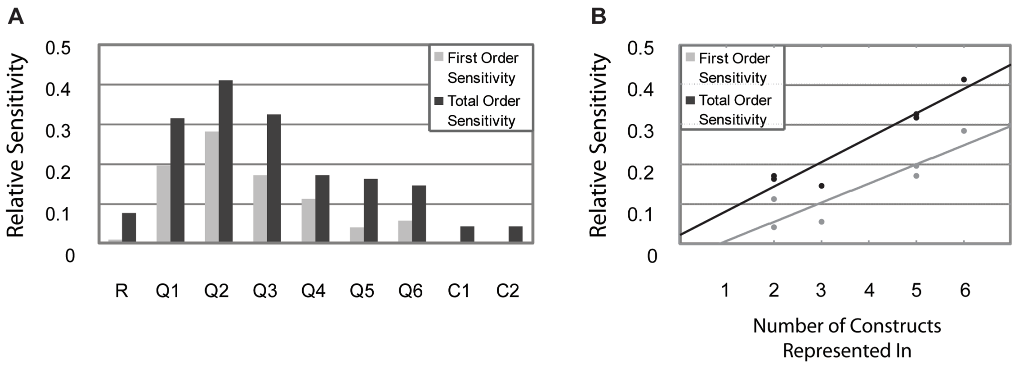

Figure S6.

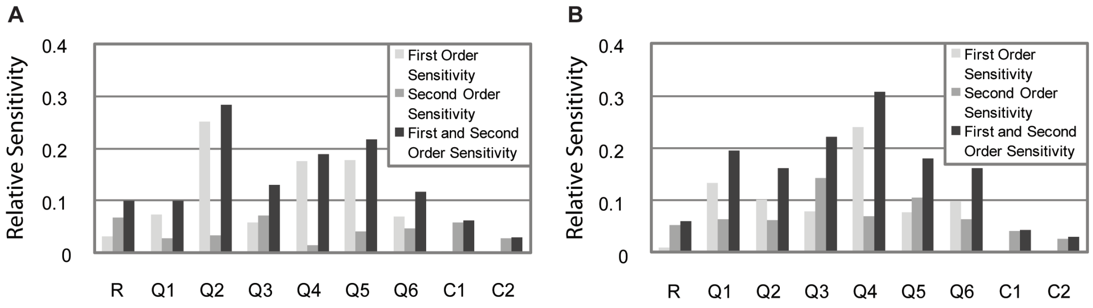

Figure S7.

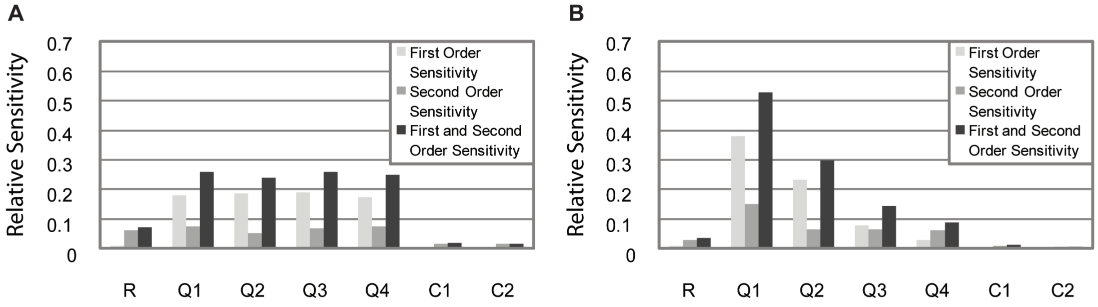

Supplement: Additional file 1 — Dresch et al. 2010. This file includes all supporting text, figures, and tables in pdf format. [file 1752-0509-4-142-S1.PDF]
